# Ranking of Sites for Installation of Hydropower Plant Using MLP Neural Network Trained with GA: A MADM Approach

**DOI:** 10.1155/2017/4152140

**Published:** 2017-02-26

**Authors:** Benjamin A. Shimray, Kh. Manglem Singh, Thongam Khelchandra, R. K. Mehta

**Affiliations:** ^1^Department of Electrical Engineering, National Institute of Technology Manipur, Manipur, India; ^2^Department of Computer Science & Engineering, National Institute of Technology Manipur, Manipur, India; ^3^Department of Electrical Engineering, NERIST, Nirjuli, India

## Abstract

Every energy system which we consider is an entity by itself, defined by parameters which are interrelated according to some physical laws. In recent year tremendous importance is given in research on site selection in an imprecise environment. In this context, decision making for the suitable location of power plant installation site is an issue of relevance. Environmental impact assessment is often used as a legislative requirement in site selection for decades. The purpose of this current work is to develop a model for decision makers to rank or classify various power plant projects according to multiple criteria attributes such as air quality, water quality, cost of energy delivery, ecological impact, natural hazard, and project duration. The case study in the paper relates to the application of multilayer perceptron trained by genetic algorithm for ranking various power plant locations in India.

## 1. Introduction

Optimal industrial site selection is an extremely complicated process as diverse and conflicting criteria are required to study in detail. Rise in industrial activity and uncontrolled use of fossil fuel has become a major cause of global warming. As a result, the climate of many places is unpredictable today and has become abnormal. The feasibility for installation of power plant is mostly location dependent which is a multi-criteria problem. Environment Impact Assessment (EIA) is invariably carried out after identifying potential site for installation of industrial plant. EIA processes act as an authoritarian requirement in site selection for over decades and have currently attracted significant research interests. Our environment which is already highly polluted will further be degraded by irresponsible and inappropriate choice of power plant installation sites. In the whole process of site selection, there exist quantifiable and epistemic uncertainties. The uncertainties associated could be modeled accurately using an algorithm that mimics natural intelligence.

During installation of industrial site like power plant, many harmful elements which are hazardous to living organism and environment will increase because of cutting down large forest area during construction phase and pollutants. Harmful gases may also be emitted due to excess fuel combustion in thermal power plant. Some of such attributes are NO_2_, which when inhaled in excess leads to respiratory problems. They may also inflame the lining of the lung and reduce immunity to lung infections. CO and SO_2_ contents must be checked as they can cause reduced oxygen delivery to body organs. Parameters such as water quality, cost of energy delivery, and construction period also play an important role in industrial power plant installation. Nonmeasurable linguistic parameters like social acceptance, willingness for resettlement, and ecological impact are the other important criteria considered in this work.

Feng developed an expert decision making system based on Rough Sets and Multiobjective Programming, for prioritizing alternative site for thermal power plant [1]. But this method could not be used as a generalized model for site selection of power plants as many of the important subattributes were not considered in the work. This finding is applicable mainly for thermal power plant.

Kaliraj and Malar use geographical information system (GIS) in identifying suitable site for thermal power plant [2]. Attributes such as land, water, coal mine, environment, settlement, and accessibility to the site were considered in the work.

Ziaei et al. used parameters like slope, land use, agriculture, and soil type for selection of industrial area applying GIS and fuzzy multi-criteria decision [3]. Nine criteria were studied which include historical and tourism centers, protected areas, slop, roads, railroads, airport, residential areas, land use, faults, and water resources. But many important criteria that are hazardous to the living organism and ecosystem were not considered.

Stoms et al. developed a fuzzy logic based model to compare the characteristics of a set of sites to study the land suitability for scientific research reserved [4]. The model combines a fuzzy logic knowledge based on specific site data obtained from GIS database. However this analysis did not account for many of the important real time scenario data as the purpose of the work is to find land suitability for scientific research. Such site selection did not have much influence on our environment.

Kengpol et al. developed a decision support system to avoid flood on solar power plant site selection by applying Fuzzy Analytic Hierarchy Process (FAHP) [5]. The results obtained through FAHP were latter verified by applying technique for order preference by similarity to ideal solution (TOPIS). In this work five main attributes, namely, climate, geographical, transportation, environmental, and cost criteria, were considered.

Sambhoo et al. applied various soft computing techniques such as backpropagation artificial neural network (BP-ANN), learning vector quantization (LVQ), fuzzy soft sets with ant colony and fuzzy indexing for ranking various power plants in India [6]. But important attributes that are related to financial aspects, social acceptance, risk management, and so forth are not considered in their work.

In this present work, multilayer perceptron-genetic algorithm (MLP-GA) is used for predicting and ranking the sites for power plant installation (both existing and upcoming) in India. The artificial neural network (ANN) in this method is trained by using a robust genetic algorithm (GA) instead of backpropagation (BP) algorithm. The current study will consider various attributes which are very much important and essentials which were not considered previously. Extensive case studies are conducted for existing as well as upcoming power plant in India. Main emphasis is given to the power plant installation in North East India as administrative irregularities, procedural violations, environmental considerations, threat of cultural extinction, lack of participatory project implementation, and absence of informed public consent of the affected were reported [7–9]. The rest of the paper is organized as follows.

Section 2 gives data of some existing and upcoming power plant in India which are considered in our case study.  Section 3 describes the proposed method.  Section 4 gives simulation results and discussions and comparison of MLP-GA and MLP-BP algorithm followed by conclusion and suggestion for future work in Section 5.

## 2. Case Study

In the case study the MLP-GA methodology is used to classify different sites for existing as well as upcoming power plants. Various criteria such as air quality, water quality, cost of energy delivery, construction period, land used, social acceptance, ecological impact, and natural hazard were considered. Altogether 19 attributes are selected for analysis. The 1500 MW Tipaimukh hydroelectric project of Manipur is a proposed site which was first proposed in 1984 but only on January 18, 2003, the project received the all-important notification under section 29 of the Electricity Act., government of India [10]. The EIA report of this power plant is not made available easily to public as there are many discrepancies. The data in our work are considered from EIA reports and the sites are located in Manipur, Arunachal Pradesh, Sikkim, and Himachal Pradesh in India [11–14]. The major attributes traditionally used in EIA study include air, water, land, and socioeconomic and ecological environment. In our work the subattributes considered for air quality assessment are NO_2_, SO_2_, PM_10_, and PM_2.5_. Nitrogen dioxide if inhaled in excess leads to respiratory problems; it can reduce immunity to lung infections. Another important pollutant considered in our study is SO_2_ which if in excess affects human health when we breathe in. It irritates the nose, throat, and airways causing coughing, wheezing, shortness of breath, or a tight feeling around the chest. For water quality assessment the subattributes considered are DO, BOD, pH value, and electrical conductivity. The water quality of the flowing water is also an important issue. Water with high amount of hardness can corrode the blades of the turbine. Also increased level of salinity can reduce the life span of the turbines. For assessing cost of energy delivery, subattributes like cost per Megawatt (MW), tariff rate, and construction period are considered. These determine both immediate and long-term effect on customer and for competitiveness of business and industry. Land used subattributes like land required per MW and land submerged per 100 MW are also considered. Other linguistic data like social acceptance, site's distance from reserved area, existence of endangered species, availability of medicinal plants, sites within seismic zone and family displaced (hostile population) and their willingness for resettlement are studied by assigning a score as per guideline given by experts around the globe and those specified by Central Pollution Control Board, 2012 [15], and Ministry of Environment and Forest (MoEF) guidelines for industries and impact assessment 201 [16]. Social acceptance or Not in My Backyard (NIMBY) impact of those selected power plants are analyzed based on different literatures survey [10, 17–19]. The ANN is trained by referring to the important subattributes in Table 1. Other linguistic subattributes mentioned earlier are also considered during the training of network.

## 3. Proposed Methodology

In this proposed methodology, backpropagation is replaced by GA to train the neural network and a correct weight for the network is obtained. Backpropagation algorithm has the drawback to become stuck at local minima and an improper selection of initial weight may delay convergence. GA, on the other hand, perform a global search, lessening the chances of becoming caught in a local minima [20]. The basic concept and various steps in formulating MLP-GA are explained in detail in the following section.

### 3.1. Multilayer Perceptron (MLP)

**Figure 1:**
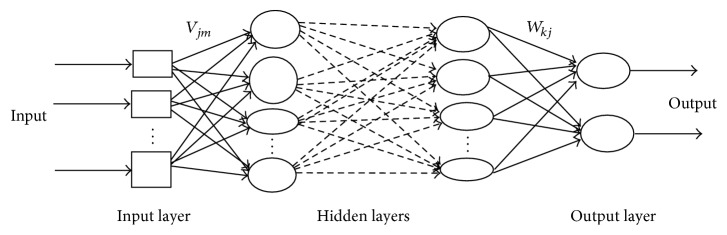
Multilayer perceptron (MLP) neural network architecture.

MLP basically composed of a supervised network, topologically configured in several layers of neurons, where each neuron of *i*th layer is connected with all neurons of (*i* + 1)th layer. The connection is implemented as a “weight,” representing the weight of the related couple of neurons. The weight is represented as a real number, usually normalized between [−1, +1]. The layers are organized into a fixed input layer, directly receiving the pattern input from user, one or more hidden layers and a fixed output layer. The hidden layers of the MLP network are considered as the brain of the network. The basic architecture of MLP network is given in Figure 1. The connection weights are determined using a training algorithm. The backpropagation learning rule popularized by Rumelhart and Mc Cleland is commonly used for training MLP network. But in our work the backpropagation learning rule is replaced by Genetic Algorithm.

### 3.2. MLP-GA Algorithm

#### 3.2.1. Initialization of the Weights

MLP is evolved by defining the genotype of the GA as the weight list. Each weight is represented as a binary number. Each solution or individual is a bit string and will represent the weights of the connections of the layers of the neural network.

In the present work, the size of each training input taken is 20. The number of hidden neuron is 4 and the number of output neuron is 1. The number of total weights (TW) is given by

(1) $$\begin{array}{c} TW=\left(\;I*HN+HN*ON\;\right), \end{array}$$

where *I* is the size of input pattern, HN is the number of hidden neurons, and ON is the number of output neurons. Therefore, the total weight in the current work is 84.

The gene length, GL, is given by the equation:

(2) $$\begin{array}{c} GL=\left[\;B*\left(\;I*HN+HN*ON\;\right)\;\right], \end{array}$$

where *B* is the number of bits per weight.

In the present work each weight is represented using 16-bit binary number, that is, *B* = 16 and hence gene length, GL = 1344.

#### 3.2.2. Reconstruction of the Phenotype from the Genotype

Consider

(3) $$\begin{array}{c} y_{m}=\overset{B}{\underset{k=1}{\sum }}b_{mk}2^{-k}, \end{array}$$

where *B* is the number of bits per weight and *b*_*mk*_ is the *k*th bit for the *m*th weight. Then,

(4) $$\begin{array}{c} w_{m}=y_{m}*A+B, \end{array}$$

where *w*_*m*_ is the weight present in the string or solution, *A* is the scaling factor, and *B* is the shifting factor.

In our application, we set *A* = 20 and *B* = −10, so that the weight will take value from [−10,10].

In this way, we get the weights *v*_*jm*_, the weight from the *m*th input to the* j*th hidden neuron, and the weights *w*_*kj*_, the weight from the* j*th hidden neuron to the* k*th output neuron.

#### 3.2.3. Output of the Hidden Layer and the Output Layer

The outputs of the hidden neurons are calculated using the relations:

(5) S1=∑m,jvjm∗xpm,

(6) yj=sigmoidS1.

Here sigmoid is a unipolar activation function.

*y*_*j*_ is the output of the* j*th hidden neuron. Calculate the output of the output neurons:

(7) S2=∑j,kwkj∗yj,

(8) ok=sigmoidS2.

*o*_*k*_ is the output of the* k*th output neuron. These two operations to find the output are performed for all the input patterns. Then the error is updated with the following:

(9) $$\begin{array}{c} E=\frac{1}{2}\overset{K}{\underset{k=0}{\sum }}{\left(\;d_{k}-o_{k}\;\right)}^{2}. \end{array}$$

*d*_*k*_ is the desired output. This process is performed until all the training samples have been used.

#### 3.2.4. Calculate the Fitness of the String or Solution

The fitness of the string or solution can be calculated using the fitness defined as

(10) $$\begin{array}{c} fitness=\frac{1-E}{N}, \end{array}$$

where *N* is the number of patterns or training samples. The above processes are repeated from Section 3.2.2 for all the strings or solutions of the population.

#### 3.2.5. Selection

Here, we find out the string with the highest fitness value. If this highest fitness value is greater than a desired fitness value (=0.99 in our application), then the operation stops. The weights representing this string with highest fitness value will be used for testing or real operation phase.

#### 3.2.6. Reproduction

The population is modified using operators, namely, crossover and mutation. The above processes from Section 3.2.2 are repeated for many generations till we get a string or solution whose fitness value is greater than the desired fitness.

## 4. Implementation

### 4.1. Results and Discussions

In this section, the results obtained after applying MLP-GA is discussed in detail. The neural network in the current study was trained by considering 19 subattributes which are grouped into five main attributes as shown in Table 1. The subattributes which are inputs to the network are classified into three classes according to some range of values as shown in Table 1. Among the input features, ecological has been given the highest weightage to calculate the ranking of the power plants. Then comes, hostile population, cost of energy delivery, water quality, and air quality in decreasing order of weightage.

The numeric data for the hydropower plants studied in our case study is given in Table 2.

After applying MLP-GA, the connection weights in the output layer are *W* = 8.139648, −0.403137, 8.547668, and −0.005188. And the connection weight in the hidden layers are *V* = 8.895874, −0.783997, −1.475525, 7.074280, 3.493652, 4.716492, 3.839722, −0.617981, −8.630371, −6.216431, −3.270874, 3.026123, −8.246155, −7.404175, 8.511047, 1.188965, −4.611206, 1.611328, 4.305115, 1.641235, −2.102356, −7.859192, −8.496704, 3.616943, −0.255737, 1.913147, 7.741394, 3.148499, 2.516785, −2.403564, 5.213013, −8.703308, −8.287048, 6.685791, −7.122192, −3.007202, −1.139832, −2.120667, −6.600952, 3.563232, −9.543457, −7.277832, −8.657227, 3.678284, −4.147034, 8.448181, −3.271179, 0.563660, −7.385559, 6.922302, 2.138672, 3.964844, −2.693176, 5.880737, 6.936340, −0.924683, −6.820068, −0.267639, 8.542480, and 4.871521.

The results obtained using MLP-GA algorithm for the case study were given in Table 3. Using MLP-GA, it is observed that Ting-Ting HEP in Sikkim is the best alternative, followed by Nafra HEP project and Bajoli HEP project. The Tipaimukh HEP of Manipur which has global controversy and faces a lot of criticism is placed as the least preferred site. More precise and accurate classifications for ranking of the power plant installation sites were achieved by increasing the generation or training cycles in GA application as illustrated in Tables 4 and 5. The ranking for the selected sites is as follows: Ting Ting HEP ranked first, Nafra HEP as second, followed by Bajoli HEP in third place, and Tipaimukh HEP ranked fourth. In our methodology the ANN is trained by using a GA instead of the most commonly used backpropagation (BP) algorithm. Backpropagation algorithm has the drawback to become stuck at local minima and an improper selection of initial weight may delay convergence but GA, on the other hand, perform a global search, lessening the chances of becoming caught in a local minima [20]. The following considerations were made for computational purpose.

Size of the input = 19, number of hidden neurons = 4, and number of output neurons = 1. The desire or target output is set to 1.0 for good, 0.05 for fair, and −1.0 for poor.

### 4.2. MLP-GA and MP-BP Results Comparison

**Figure 2:**
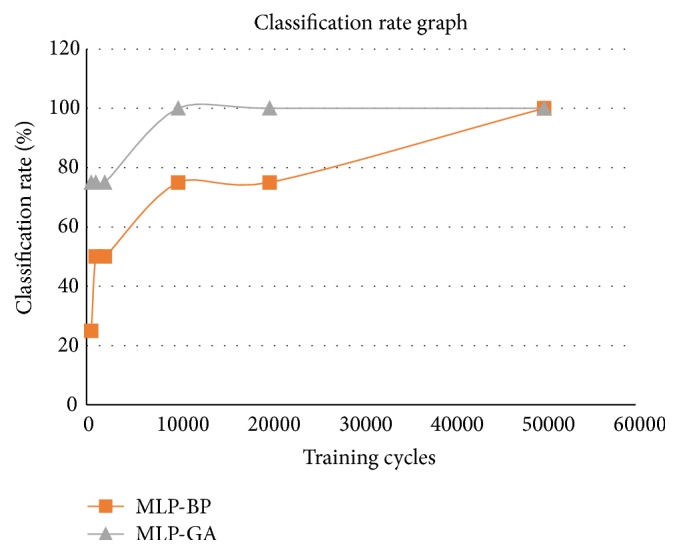
Graph showing MLP-GA versus MLP-BP performance.

The results obtained after training MLP by GA is compared with results obtained after training MLP by BP algorithm. The results of comparison for two training cycles, that is, 2000 training cycles and 10000 training cycles, are shown for illustrative purpose in Tables 4 and 5, respectively. More in-depth analysis and comparisons for different iterations or training cycles are illustrated graphically in Figure 2.

*(i) Comparison for 2000 Training Cycles*

*(a) MLP-BP.* The connection weights in the output layer* “W”* are as follows: 0.734139, 2.041600, 0.617518, 2.097973, 6.432362, −2.556959, 0.336022, −2.384376, 0.425262, 2.518832.

The connection weights in the hidden layer* “V”* are as follows: 0.305531, −0.450689, 0.428237, 0.330757, 0.365426, −0.389095, 0.414114, −0.420629, −0.136728, −0.289917, 0.431811, 0.087642, 0.131555, 0.223131, −0.471790, −0.343707, −0.164725, 0.400936, −0.296919, 0.453303, 0.308819, 0.170333, 0.234527, −0.063943, 0.285621, −0.047045, −0.026247, 0.397211, −0.246425, −0.365713 0.290721, −0.314819, 0.119038, 0.246225, 0.483102, −0.269123, 0.183132, −0.208129, −0.100440, 0.136836, −0.211080, −0.076677, 0.402333, 0.265095, 0.473855, 0.414572, 0.038468, −0.389990, 0.139772, 0.032763 −0.439788, 0.181634, −0.399938, 0.174647, −0.416758, −0.031811, 0.211765, 0.169842, 0.365121, 0.184724, 0.487932, −0.264314, 0.467117, 0.358658, 0.016569, −0.387160, −0.432766, 0.119724, 0.088440, −0.390428, −0.089655, −0.078576, −0.217386, 0.387006, 0.393366, 0.474731, −0.478409, −0.257644, −0.130047, 0.329742. 

*(b) MLP-GA*. The connection weights in the output layer are as follows: 8.640137, −0.108032, −6.368103, −2.590332, 0.653076, −7.587585, −3.596191, −5.078430, 9.705811, 7.773438.

The connection weights in the hidden layer are as follows: −0.362244, 8.981323, −0.627136, −1.430359, 5.731201, 5.973511, −1.858521, −0.414429, 8.376160, −2.780762, 2.784119, 9.869385, −6.218567, −1.531677, 4.980164, 9.441223, −1.383057, 0.783386, 1.921082, 1.910706, 3.632813, 7.511902, −1.937866, 7.555847, 4.084473, 3.894348, 2.620544, −9.647217, −6.548462, 9.605713, 0.293579, −9.856873, 2.045288, 6.323853, 7.258301, −6.036377, −5.184631, 7.230835, −6.697998, 6.506653, 3.273315, −3.641663, 6.458435, 3.102722, −8.251343, −8.585510, −2.097168, −0.351257, 4.871216, 6.976013, −0.329590, −3.218994, 7.872925, −1.158752, −9.348145, 5.022583, −3.162537, −0.123901, 3.478394, −7.898560, 0.076904, 4.951477, −4.499207, −3.388062, 5.930786, 4.740906, 6.850281, 3.627014, −5.051270, −4.497681, −6.159363, −3.559570, 2.778015, −6.582642, −3.999329, −3.980713, −5.700073, −4.452515, 4.456177, 1.010437.

The following considerations were made for simulation purpose.

Size of the input = 19, number of hidden neurons = 5, and number of output neurons = 2. The desire or target output is set to {1,0} for good, {0,1} for fair, and {0,0} for poor.

*(ii) Comparison for 10000 Training Cycles*

*(a) MLP-BP.* The connection weights in the output layer* “W” *are as follows: 0.188078, 0.492327, 0.574531, −4.225360, 3.730163, 1.013063, 0.378569, 0.476830, −4.618770, 3.839934.

The connection weights in the hidden layer* “V” *are as follows: −0.461304, −0.062544, −0.021948, 0.408009, −0.453905, 0.174533, −0.179232, 0.287021, 0.142736, −0.170833, −0.298220, −0.044246, 0.152835, −0.293921, 0.354115, −0.034447, −0.288021, −0.311719, −0.378712, 0.116438, 0.469377, −0.026979, −0.178748, −0.101630, −0.066713, −0.137334, −0.300564, 0.498599, −0.079203, 0.234070, −0.427677, 0.244691, −0.051125, 0.310323, 0.306384, 0.207389, −0.480142, 0.180512, 0.163735, 0.411557, −0.336028, −0.148515, 0.238227, 0.050782, 0.486782, −0.155961, 0.496837, 0.432305, 0.457947, 0.352271, −0.300943, 0.044913, 0.116874, −0.237545, 0.478014, 0.235534, −0.327268, −0.080950, −0.466530, −0.470620, 0.065543, 0.079142, −0.330717, −0.265523, 0.334417, 0.155434, −0.171933, −0.444706, 0.366013, −0.318618, −0.258024, 0.215928, 0.377712, 0.279822, −0.306119, −0.227227, −0.369113, 0.299920, 0.278122, 0.371013.

*(b) MLP-GA.* The connection weights in the output layer* “W” *are as follows: −1.679993, −6.200562, 4.992676, −0.397034, 0.607910, −4.313660, 7.661743, 4.687500, −1.875000, 7.633972.

The connection weights in the hidden layer* “V” *are as follows: 4.173584, −7.783203, 8.825989, 3.720093, −5.450439, −3.009949, −7.048645, −2.969971, −8.653870, 0.428162, −6.829529, 6.887207, −8.811035, 5.026855, −8.310547, 4.942932, −0.180969, 2.826538, 5.613403, 8.455811, 8.414612, 7.419434, −6.508484, −1.716309, 6.739502, −4.996948, −5.166321, 0.060425, −2.833557, 1.747437, −1.610718, −1.657410, 1.234436, 5.168152, −7.507935, −7.496033, 4.292603, −6.580505, 8.094482, 3.653870, −8.523560, 8.390808, 5.028381, −3.546448, −2.420959, 9.117432, −9.519043, −0.023499, −2.316895, 0.456848, 6.387634, 0.820923, −5.253906, −2.333679, 9.919434, 9.270020, 0.936279, 7.049866, 5.543518, −5.120239, 8.855896, −2.654419, −6.263733, −6.665039, −8.846130, 9.176941, −2.744446, 1.533508, 3.603210, −0.364990, 1.494751, 8.595886, 4.587402, 4.457092, −0.513000, 6.563721, −6.754456, −2.308350, −3.476868, −4.787598.

The following considerations were made for simulation purpose.

Size of the input = 19, number of hidden neurons = 5, and number of output neurons = 2. The desire or target output is set to {1,0} for good, {0,1} for fair, and {0,0} for poor.

The comparisons in Tables 4 and 5 show that the proposed MLP trained with GA performs better than MLP trained with conventional BP algorithm. It is also observed that MLP-GA could accurately classify and rank power plant installation with lesser training cycle. Figure 2 shows the detailed analysis of MLP-GA and MLP-BP for different learning cycles or iterations.

The *x*-axis represents the learning cycles or iterations while *y*-axis represents the percentage classification rate. The percentage classification rate that gives ranking of sites for hydropower plant installation is studied for 500, 1000, 10000, 20000, 50000, and 60000 learning cycles or iterations for the newly proposed MLP neural network trained by GA and MLP neural network trained by BP algorithm. For the case study, the computational evaluation shows that, for 500 learning cycles, MLP-GA classification rate is 75% but, for MLP-BP, the percentage classifications rate is only 25%. In a similar pattern, it is found that starting from 10000 learning cycles, MLP-GA's attained 100% classification rate success; that is, it can precisely rank the given power plant installation sites. But MLP-BP classification rate is only 50% at 2000 iterations and 75% at 10000 to 20000 iterations. MLP-BP attains 100% classification rate only after reaching 50000 iterations. As such, the proposed methodology of training of MLP neural network by GA shows much higher efficiency in accurately classifying and identifying potential sites for installation of hydropower plants.

## 5. Conclusion and Future Work

Real world decision making regarding site selection for installation of hydropower plant is a complex issue and needs careful analysis as it involves the participation of all the stakeholders including a common man. Hydropower plant installation involves heavy financial investment, manpower, and time constraints, thereby turning it into an almost irreversible decision after its installation. Therefore, a full-proof method to avoid harmful effects to the environment and subsequently to mankind is required. The location of hydropower plants becomes a debatable issue in country where there is huge demand to meet the ever increasing energy needs. Many policy makers may attempt to tap power without considering the ill effect properly which may be a threat to our environmental ecosystem and human existence. Since the problem of site selection involves quantitative as well as qualitative attributes which must sometimes be described with linguistic Tinformation, ANN based formalism seems to be more suitable to address the problem. The proposed MLP-GA shows that it can accurately prioritize potential sites for hydropower plants installation. Our results are unbiased in nature and different important criteria, both quantitative and qualitative information about the hydropower plant sited, were considered.

Attributes relevant to the process of choosing a venture like using capacity factor (CP), internal return rate (IRR), and systemic benefits may be discussed later on considering the business world requirement but in our current study our objective is to consider environmental impact which is very much important to sustain. The synergy of neural network combined with fuzzy logic for ranking of sites for installation of hydropower plant will form extension to the work discussed in this paper.

## Figures and Tables

**Table 1:** Data used in training the neural network.

Attributes	Subattributes	Class good	Class fair	Class poor
Air quality	SO_2_ (*µ*g/m^3^)	0 < *x* < 35	36 ≤ *x* ≤ 70	*x* > 70
	NO_2_ (*µ*g/m^3^)	0 < *x* < 40	41 ≤ *x* ≤ 70	*x* > 70
	PM_2.5_ (*µ*g/m^3^)	0 < *x* < 20	21 ≤ *x* ≤ 55	*x* > 55
	PM_10_ (*µ*g/m^3^)	0 < *x* < 40	41 ≤ *x* ≤ 70	*x* > 75

Water quality	Ph	0 < *x* < 5.5	5.6 ≤ *x* ≤ 8	*x* > 8
	DO (mg/l)	*x* > 20	7 ≤ *x* ≤ 20	*x* < 7
	BOD (mg/l)	0.1 < *x* < 1.5	1.6 < *x* < 3.0	3.1 < *x* < 10
	Electrical conductivity (*µ*mhos/cm)	0 < *x* < 1500	1500 ≤ *x* ≤ 2100	*x* > 2100

Cost of energy delivery	Cost/MW (Cr)	0 < *x* < 1.5	1.5 < *x* < 4.5	*x* > 4.5
	Tariff rate (Rupee)	0 < *x* < 2.0	2.0 ≤ *x* ≤ 4.0	*x* > 4.0
	Construction period/100 MW (Yr)	0 < *x* < 3.0	3.0 ≤ *x* ≤ 4.0	*x* > 4.0

Hostile population	Family affected/100 MW (numbers)	0 < *x* < 30	30 ≤ *x* ≤ 70	*x* > 70
	Social acceptance (score)	0 < *x* < 7	7 ≤ *x* ≤ 15	*x* > 15
	Seismicity (score)	*x* > 20	7 ≤ *x* ≤ 20	0 < *x* < 5

Ecological	Endangered species presence (score)	*x* > 15	7 ≤ *x* ≤ 15	0 < *x* < 7
	Medicinal plant presence (count)	0 < *x* < 4	4 ≤ *x* ≤ 10	*x* > 10
	Presence of national park/reserved area (within KM)	*x* > 60	30 ≤ *x* ≤ 60	0 < *x* < 30
	Land required (ha)/MW	0 < *x* < 1.5	1.5 ≤ *x* ≤ 2.7	*x* > 2.7
	Land submerged (ha)	0 < *x* < 1	1 ≤ *x* ≤ 2.5	*x* > 2.5

**Table 2:** Numeric data for the power plant taken in the case study.

Attributes	Subattributes	Site 1 (Bajoli HEP, Himachal Pradesh) (180 MW)	Site 2 (Ting Ting HEP, Sikkim) (97 MW)	Site 3 (Tipaimukh HEP, Manipur) (1500 MW)	Site 4 (Nafra HEP, Arunachal Pradesh) (96 MW)
Air quality	SO_2_ (*µ*g/m^3^)	7.1	6.80	80	50
	NO_2_ (*µ*g/m^3^)	4.4	9.0	80	24
	PM_2.5_ (*µ*g/m^3^)	44	19.0	60	21
	PM_10_ (*µ*g/m^3^)	105	79.0	100	56

Water quality	Ph	7.30	7.19	7.5	7.0
	DO (mg/l)	9.60	8.81	2.9	5.0
	BOD (mg/l)	9.0	3.0	8.4	3.0
	Electrical conductivity (*µ*mhos/cm)	3700	44.33	2200	2350

Cost of energy delivery	Cost/MW (Cr)	10.42	4.91	5.43	7.19
	Tariff rate (Rupee)	5.05	2.22	4.65	3.55
	Construction period/100 MW (Yr)	5.0	2.5	7.5	3.0

Ecological	Land required (ha)/MW	0.522	0.313	6.67	0.780
	Land submerged (ha)	2.0	1.02	3.5	2.3

**Table 3:** Results obtained by applying MLP-GA.

Power plant site	Test input (*x*_*pm*_) *(the 19 subattributes which are used for each plant)*	Output from hidden layer *y*_*j*_ = sigmoid (*s*_1_) where *s*_1_ = *v*_*jm*_*∗x*_*pm*_	Output from output layer *o*_*k*_ = sigmoid (*s*_2_) where *s*_2_ = *w*_*kj*_*∗y*_*j*_	Rank
(1) Bajoli HEP, Himachal Pradesh (180 MW)	98.0, 80, 60, 140, 7.30, 9.60, 9.0, 3700, 10.42, 5.05, 5.0, 0.522, 50.00, 15.00, 11.0, 5.00, 50.0, 10.0, 2.0, −1	*y*[0] = −1.000000	0.050311	II
		*y*[1] = 1.000000		
		*y*[2] = 1.000000		
		*y*[3] = −1.00000		

(2) Ting Ting HEP, Sikkim (97 MW)	6.80, 9.00, 19.00, 79.0, 7.19, 8.81, 3.00, 44.33, 4.91, 2.22, 2.50, 0.313, 10.00, 2.00, 7.00, 5.00, 5.00, 15.00, 1.02, −1	*y*[0] = 1.000000	1.000000	I
		*y*[1] = 1.000000		
		*y*[2] = 1.000000		
		*y*[3] = −1.00000		

(3) Tipaimukh HEP, Manipur (1500 MW)	80, 80, 60, 100, 7.5, 2.9, 8.4, 2200, 5.43, 4.65, 7.5, 6.67, 291, 20, 30, 5.0, 20, 10.0, 3.5, −1	*y*[0] = −1.000000	−1.000000	III
		*y*[1] = 1.000000		
		*y*[2] = −1.00000		
		*y*[3] = −1.00000		

(4) Nafra HEP, Arunachal Pradesh (96 MW)	50, 24, 21, 56, 7.0, 5.0, 3.0, 2350, 7.19, 3.53, 3.0, 0.780, 5, 1, 0, 10, 4, 4, 2.3, −1	*y*[0] = −1.000000	0.050311	II
		*y*[1] = 1.000000		
		*y*[2] = 1.000000		
		*y*[3] = −1.00000		

**Table 4:** Results' comparison for 2000 training cycles or iterations.

Sl. no.	Power Plant	Number of learning cycles/iterations	MLP-BP		MLP-GA	
			Output from output layer	Rank	Output from output layer	Rank
(1)	Bajoli HEP, Himachal Pradesh	2000	0.091722, 0.147098	II	0.000000, 1.000000	II
(2)	Ting Ting HEP, Sikkim		0.280671, 0.280671	I	1.000000, 0.000000	I
(3)	Tipaimukh HEP, Manipur		0.091722, 0.147098	II	0.000000, 0.000000	III
(4)	Nafra HEP, Arunachal Pradesh		0.091722, 0.147098	II	0.000000, 1.000000	II

**Table 5:** Results' comparison for 10000 training cycles or iterations.

Sl. no.	Power Plant	Number of learning cycles/iterations	MLP-BP		MLP-GA	
			Output from output layer	Rank	Output from output layer	Rank
(1)	Bajoli HEP, Himachal Pradesh	10000	0.000613, 0.122231	III	0.000000, 0.000030	III
(2)	Ting Ting HEP, Sikkim		0.777613, 0.002231	I	1.000000, 0.000000	I
(3)	Tipaimukh HEP, Manipur		0.000613, 0.122231	III	0.000000, 0.000000	IV
(4)	Nafra HEP, Arunachal Pradesh		0.000613, 0.722231	II	0.000000, 0.007000	II

## References

[1] Feng R. Optimal site selection for thermal power plant based on rough sets and multi-objective programming.

[2] Kaliraj S., Malar V. K. (2012). Geospatial analysis to assess the potential site for coal based thermal power station in Gujarat, India. *Advances in Applied Science Research*.

[3] Ziaei M., Hajizadeh F., Ahmadizadeh S. S. R., Jahanifar K. (2012). A combined model of GIS and fuzzy multi criteria decision analysis for suitable evaluation/selection of industrial areas. *Recent Researches in Environmental Science and Landscaping*.

[4] Stoms D. M., McDonald J. M., Davis F. W. (2002). Fuzzy assessment of land suitability for scientific research reserves. *Environmental Management*.

[5] Kengpol A., Rontlaong P., Tuominen M. (2013). A decision support system for selection of solar power plant locations by applying fuzzy AHP and TOPSIS: An Empirical Study. *Journal of Software Engineering and Applications*.

[6] Sambhoo K., Kadam S., Deshpande A. (2014). Ranking of sites for power plant installation using soft computing techniques—a thought beyond EIA. *Applied Soft Computing*.

[7] Arora V., Kipgen N. (2012). We can live without power, but we can't live without our land: indigenous hmar oppose the Tipaimukh Dam in Manipur. *Sociology Bulletin*.

[8] Yumnam J., Koijam P. Tipaimukh Dam Plan and Uncertainties in Manipur. http://cramanipur.blogspot.in/p/article.html.

[9] Hossain M. A. http://archive.riversymposium.com/2005/index.php?element=06HOSSAIN+MAnowar.

[10] Ranjan R. K. (2003). Tipaimukh. *The Ecologist Asia*.

[11] Envirolink Technologies R. S., TT Energy (2010). *Environmental Impact Assessment of H.E. Project*.

[12] Envirolink Technologies R. S. (2010). GMR Bajoli Holi hydro power plant. *Environmental Impact Assessment Report*.

[13] Smec India SEW Nafra Corporation. Environmental Impact Assessment and Environmental Management Plan for NAFRA Hydro Electric Power Project. http://www.moef.nic.in/sites/default/files/Report-I.pdf.

[14] Agricultural Finance Corporation Ltd (2007). *Comprehensive Environmental Studies on Tipaimukh Hydro-Electric (Multi-Purpose) Project*.

[15] Central Pollution Control Board, Government of India, 2012, http://www.cpcb.nic.in/upload/NewItems/NewItem_192_NAAQSTI.pdf

[16] Ministry of Environment and Forest (MoEF) (2011). *Guide Lines for Industries and Impact Assessment*.

[17] Objections to forest clearance of 180 MW bajoli-holi hydro-electricity project in himachal pradesh. http://www.himdhara.org/2012/11/16/objections-to-forest-clearance-to-180-mw-bajoli-holi-hydro-electricity-project-in-himachal-pradesh/.

[18] Huber A., Joshi D. (2015). Hydropower, anti-politics, and the opening of new political spaces in the Eastern Himalayas. *World Development*.

[19] Islam M. S., Islam M. N. (2016). ‘Environmentalism of the poor’: the Tipaimukh Dam, ecological disasters and environmental resistance beyond borders. *Bandung: Journal of the Global South*.

[20] Larose D. T. (2006). *Data Mining Methods and Models*.

